# Molecular Dynamics Simulations to Decipher the Role of Phosphorylation of SARS-CoV-2 Nonstructural Proteins (nsps) in Viral Replication

**DOI:** 10.3390/v14112436

**Published:** 2022-11-02

**Authors:** Lamya Alomair, Sabeena Mustafa, Mohsin Saleet Jafri, Wardah Alharbi, Abdulrhman Aljouie, Fahad Almsned, Mohammed Alawad, Yahya Abdulfattah Bokhari, Mamoon Rashid

**Affiliations:** 1 King Abdullah International Medical Research Center (KAIMRC), King Saud bin Abdulaziz University for Health Sciences (KSAU-HS), Ministry of National Guard Health Affairs (MNGHA), Riyadh 11426, Saudi Arabia; 2 School of Systems Biology and the Krasnow Institute for Advanced Study, George Mason University, Fairfax, VA 22030, USA; 3 Center for Biomedical Engineering and Technology, University of Maryland School of Medicine, Baltimore, MD 21201, USA; 4 Research Center, King Fahad Specialist Hospital in Dammam (KFSH-D), Dammam 32253, Saudi Arabia; 5 Population Health Management, Eastern Health Cluster, Dammam 32253, Saudi Arabia; 6 Research and Development Department, Novo Genomics, Riyadh 12271, Saudi Arabia

**Keywords:** nonstructural proteins, phosphorylation, post-translational modifications, kinases, molecular dynamics

## Abstract

Protein phosphorylation is a post-translational modification that enables various cellular activities and plays essential roles in protein interactions. Phosphorylation is an important process for the replication of Severe Acute Respiratory Syndrome Coronavirus 2 (SARS-CoV-2). To shed more light on the effects of phosphorylation, we used an ensemble of neural networks to predict potential kinases that might phosphorylate SARS-CoV-2 nonstructural proteins (nsps) and molecular dynamics (MD) simulations to investigate the effects of phosphorylation on nsps structure, which could be a potential inhibitory target to attenuate viral replication. Eight target candidate sites were found as top-ranked phosphorylation sites of SARS-CoV-2. During the process of molecular dynamics (MD) simulation, the root-mean-square deviation (RMSD) analysis was used to measure conformational changes in each nsps. Root-mean-square fluctuation (RMSF) was employed to measure the fluctuation in each residue of 36 systems considered, allowing us to evaluate the most flexible regions. These analysis shows that there are significant structural deviations in the residues namely nsp1 THR 72, nsp2 THR 73, nsp3 SER 64, nsp4 SER 81, nsp4 SER 455, nsp5 SER284, nsp6 THR 238, and nsp16 SER 132. The identified list of residues suggests how phosphorylation affects SARS-CoV-2 nsps function and stability. This research also suggests that kinase inhibitors could be a possible component for evaluating drug binding studies, which are crucial in therapeutic discovery research.

## 1. Introduction

### 1.1. History, Transmission, and Epidemiology

Severe Acute Respiratory Syndrome Coronavirus 2 (SARS-CoV-2) is a positive-stranded RNA virus that causes severe acute respiratory syndrome in humans [1]. The World Health Organization (WHO) declared Coronavirus Disease-2019 (COVID-19) a severe pandemic, infecting many people worldwide. A better understanding of coronavirus biology has evolved after extensive research on human coronaviruses. Many coronaviruses are estimated to have crossed from animal hosts to humans [2]. The SARS-CoV, MERS-CoV, and SARS-CoV-2 observed in bats is thought to be then transmitted to other animals across diverse geographical areas [3,4]. The coronaviruses belong to the family Coronaviridae and are classified into; alpha, beta, gamma, and delta coronaviruses (α-CoV, β-CoV, γ-CoV, and δ-CoV, respectively) [5]. The beta coronaviruses are classified into diverse lineages (A, B, C, and D); both SARS-CoV and SARS-CoV-2 belong to lineage B, while MERS-CoV is classified in lineage C. Phylogenetic analysis confirms that the SARS-CoV-2 proteins originated in the bat coronavirus’s beta-genus lineage [6].

Compared to SARS-CoV, SARS-CoV-2 is more infectious and transmissible [7]. The SARS-CoV-2 incubation period is estimated between 1–14 days, with a median incubation of 4 days [8]. Generally, the symptoms include fever, cough, diarrhea, fatigue, and difficulty breathing, similar to SARS-CoV infection symptoms [9]. The human-to-human infections of SARS-CoV-2 also occur from infected people or an asymptomatic carrier [10].

### 1.2. SARS-CoV-2 Genome and Proteome

The viral genome structure and its replication mechanisms are crucial to understand the pathogenic cycle of SARS-CoV-2 and to develop potential therapeutic options. Among all RNA viruses, coronaviruses are considered one of the largest genomes ranging from 26–32 kb in length. The genome of SARS-CoV-2 measures 80% similarity with the SARS-CoV genome and 96% similarity with bat coronavirus BatCoV-RaTG13 [10]. Structurally, the SARS-CoV-2 genome consists of five major open reading frames (ORFs); these contain a nonstructural 5′ polyprotein (ORF1a and ORF1ab) and four canonical 3′ structural proteins, known as spike protein (S), an envelope protein (E), a membrane protein (M), and a nucleocapsid protein (N) [11]. The SARS-CoV-2 genome has ten ORFs classified into two groups: ORF1ab and ORFs 2–10 [12]. ORF1ab encodes the replicase polyprotein 1ab, with various functions in the virus’s RNA transcription and replication. The structural proteins of the virus, such as S, E, M, and N proteins, are encoded by ORFs 2–10, which are essential in RNA genome assembly [13]. The PLpro cuts three N-terminal sites, and the 3CLpro cuts 11 C-terminal sites, resulting in 16 nsps. The helical capsid (N) protein accumulates the viral RNA genome, while the viral lipid envelope is formed by the S, E, and M proteins [14]. The viral genome requires both envelope and membrane proteins for assembly, whereas the spike protein is required for host cell recognition and virus endocytosis [15].

### 1.3. Pathogenicity Mechanism of SARS-CoV-2

Several processes are involved, from the SARS-CoV-2 viral entry to the cell and virus replication cycle. Generally, viral RNA and protein hijack the host cell’s mechanism to create the replication–transcription complex to initiate the genome replication of virus and polypeptide chain formation necessary to synthesize the virus RNAs’ subgenome and the structural proteins [16]. Each step of this mechanism includes many viral proteins and occurs in a diverse location in the host cell. Recent research shows that SARS-CoV-2 follows the nidovirus pattern of infection, which includes seven domains [17]. Two domains initiate from nsp3, the ADRP-domain (ADP-ribose phosphatase) and the PLpro-domain (papain-like protease). The ADRP showed a crucial role in the cell signaling process to inhibit the host immune response [18].

The main protease (MPro), nsp5, is necessary for the viral polypeptide cleavage into its functional proteins, and the new RNA virus production will be facilitated by RNA-dependent RNA polymerase (RdRp), nsp12. Both viral proteases have been shown to inhibit the human immune system by interacting with SARS-CoV-2 immune proteins [19]. Additionally, the SARS-helicase sequence, nsp13, is conserved and essential, being a fundamental part of the replication mechanism of coronaviruses. Furthermore, the nsp15, a highly conservative uridine-specific endoribonuclease, affects the viral RNA replication by inhibiting the host’s innate immune response. It has been reported that viruses have evolved to use the process of phosphorylation by host-cell kinases to enhance replication and inhibit normal cellular functions [20].

### 1.4. Phosphorylation, Its Importance in Replication of Viral Proteins, and Scope of This Research

Phosphorylation plays a major role in many cellular processes, requires a human protein kinase as part of post-translational modification events, and produces conformational modifications that lead to functional changes [21]. In SARS-CoV-2, the role of phosphorylation of specific nsps in replication is still poorly understood. On the other hand, phosphorylation of viral proteins of different viruses has been extensively studied [22]. Earlier studies detected around 49 phosphorylation sites in SARS-CoV-2 viral proteins. These predicted kinase groups could contribute to control the viral replication and regulate those phosphorylation sites involved casein kinase II (CKII), cyclin-dependent kinase (CDK), and protein kinase C (PKC). Although it is doubtful that all phosphorylation sites on the proteins of virus play a key functional role, numerous sites in (M) protein, nsp9, and (N) proteins, as examples, have been suggested to have a functional role [23]. Computational and proteomics studies of phosphorylation mechanisms have a significant role in elucidating viral pathogenicity [24]. First, this research project employs computational methods to study protein–protein interactions between SARS-CoV-2 proteins and human protein kinase to identify potential phosphorylation sites. The phosphorylation sites will be evaluated to investigate our hypothesis by performing molecular dynamics (MD) simulation analysis of conformational modifications that lead to functional changes then prioritizing and identifying a set of residues that take part in the phosphorylation process of SARS-CoV-2 in order to facilitate the identification of specific targets, and to understand the system behavior thereby helps to identify potential therapeutic targets for further experimental work. Also, based on understanding the essential role of nsps, we have chosen them for our MD simulations study. We present a pipeline of computational methods to investigate the functional role of phosphorylated proteins at the atomic level. MD simulation studies are highly sophisticated and computationally expensive. Thus, we combined computational methods and biochemical approaches to understand better how phosphorylation events of SARS-CoV-2 nsps affect their structure and function.

## 2. Materials and Methods

### 2.1. Sequence and Crystal Structure Retrieval

**Figure 1:**
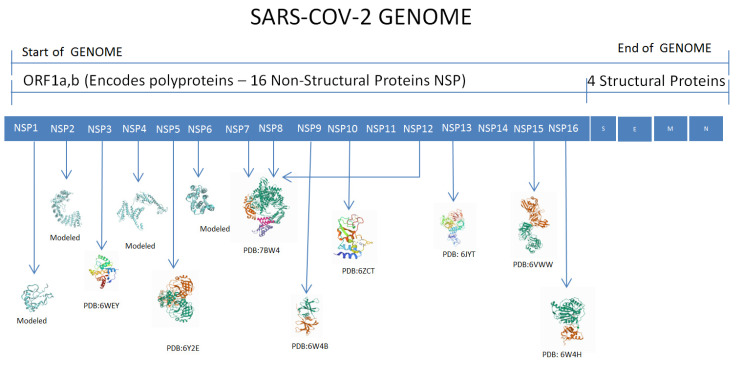
Cartoon representation of the SARS-CoV-2 nonstructural proteins (nsps).

**Figure 2:**
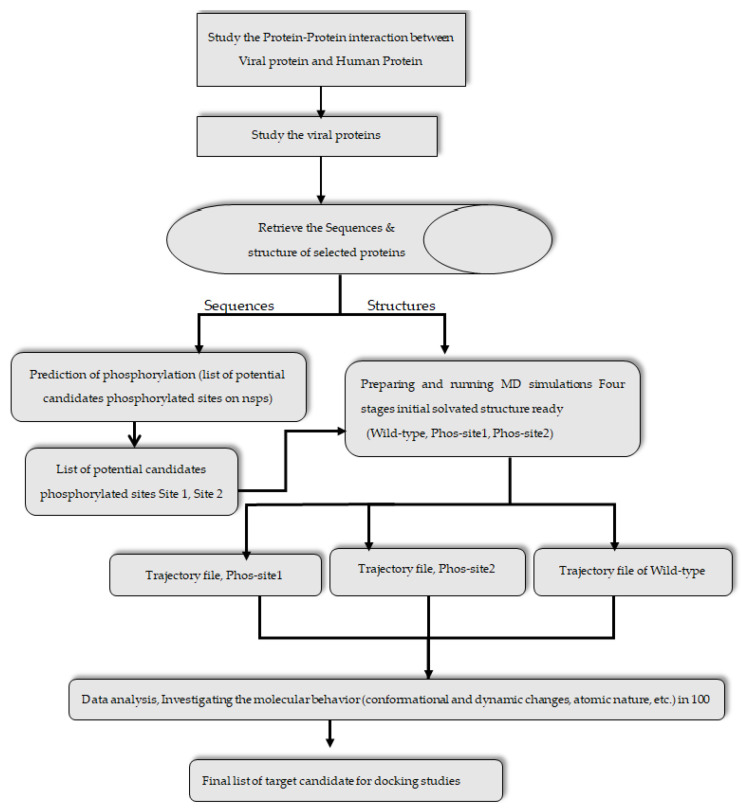
Pictorial representation of various methodologies followed in this study.

Figure 1 shows SARS-CoV-2 and the 12 nsps. These 12 nsps sequences and their structures were considered for phosphorylation site prediction. The Protein Data Bank (PDB) provided the wild-type (WT) crystal structures used in these studies. Swiss Model Webserver (https://swissmodel.expasy.org/ (accessed on 10 September 2020) was used to model the structure of nsps which are not available in PDB. Figure 2 shows the pictorial representation of the methods followed.

Twelve structures were selected for this study. Nsp1 consists of 107 amino acids, and nsp2 consists of 638 amino acids modeled using a Swiss Model Webserver. Nsp3 structure was retrieved as a high-resolution structure macro X domain (PDB- 6WEY) which consists of 172 amino acid residues. The nsp3 is one of the largest proteins in the coronavirus genome, with a molecular weight of about 200 kD [25].The initial structure of nsp4 was modeled using the Swiss Model Webserver, Nsp4, which has 500 amino and it plays a fundamental role in nucleate and anchor viral replication complexes [26]. The PDB crystal structure of nsp5, the free enzyme of the SARS-CoV-2 main protease (PDB: 6Y2E), contains 306 amino acids. Nsp6 consists of 290 amino acids that were modeled by the Swiss Model Webserver. In the case of nsp12–7-8 studies, the RNA-dependent RNA polymerase selected consists of 923 amino acids (PDB: 7BW4).

### 2.2. Prediction of Phosphorylation

Three online servers, which have the most reliable algorithms, were used to identify possible amino acid residues in nsps that might undergo phosphorylation by human kinase proteins. GPS 3.0 server is the first tool selected for the analysis [27,28]. The second prediction tool was NetPhos, whose algorithm is based on position-specific scoring matrices and artificial neural networks, and the third tool is Scansite, which is based on position-specific scoring matrices [29]. The sequences of the 12 nsps were submitted in FASTA format. The results were interpreted based on the highest scores and the consensus region identified by all three tools using a prediction score cutoff (GPS > 9, NetPhos > 0.4, and Scansite > 0.4). Two phosphorylation sites for each nsps that showed a value over the threshold were chosen for further processing into phosphorylated structures. A total of 24 sites were considered for further investigation. To generate phosphorylated models, we use an SP2 phosphoserine patch for serine and a THP2 phosphoserine patch for threonine, which both have a charge of −2.

### 2.3. Solvation of 36 Systems

All twelve WT structures and the 24 phosphorylated structures of nsps (total 36 systems) were solvated in a cubic periodic box with a TIP3P water model with a minimum distance from the protein surface spanning 15 Å from the system. A water model is essential in computational chemistry, which is generally used to prepare simulations to determine the water clusters, liquid water, and solutions with a solvent. An important aspect of using this water model is related to quantum and molecular mechanics [30]. This also helps to justify clarity in investigating the simulation process’s structural, energetic, and dynamic nature. As a next step, ions were added to neutralize the net charge of the systems and set the ion concentration to b 0.15 Mol/L. Detailed descriptions of the number of atoms and water box dimensions are shown in Table 1.

### 2.4. Preparing Molecular Dynamics (MD) Simulations

MD simulation is a computational method for studying the physical movement of atoms and molecules at the atomic level using computer techniques. The MD Simulation was performed by using Nanoscale Molecular Dynamics (NAMD2) with CHARMM36 all-force field parameters parallel programming model. Periodic boundary conditions were used to simulate the structures reported every 2 ps. We used a 12 Å cutoff for van der Waals interaction with a switching function distance of 8 Å, and the smooth particle-mesh Ewald (PME) method was enabled accordingly. The simulations of the 36 systems were prepared as follows: They started with systems energetically minimized to adjust the structure force field and relaxed possible steric clashes to obtain a low-energy starting conformation. To avoid distress, a total of 100 steps of minimization were performed. The system then was heated from 0 to normal physiological conditions up to 310 K for 300 ps, with the Langevin thermostat applied. The 36 systems were simulated to sample the structural characteristics and dynamics at 300 K for 100 ns and time step 2 fs. The long-range electrostatics were handled with the particle-mesh Ewald (PME) methods. We used NAMD software (University of Illinois at Urbana–Champaign), with GPU acceleration to speed up computation, NAMD version 3.0, Linux multicore GPU 159 NVIDIA CUDA acceleration capability. KAIMRC, HPC, Riyadh, Saudi Arabia was used to perform all simulations had the following configuration: CPU: Intel Xeon gold 6126 (12 cores) @ 2.60 GHz, 161 Memory (RAM): 196 GB, GPU: Tesla V100 (VRAM 32GB).

### 2.5. Trajectories Analysis

MD simulations of the 36 systems (WT, Phosphorylated site 1, Phosphorylated site 2) were performed for 100 ns using NAMD at 300 K. Periodic boundary conditions were used with structures reported every 2 ps, in order to study the structural effects of the phosphorylation on the nsps structures. The root mean square devotion (RMSD) and root mean square fluctuation (RMSF) were calculated for all trajectory structures, using R package Bio3D to identify the overall binding structure differences.

The Bio3D R package (v 2.4-1) was used to calculate the overall deviation from the initial structure, Root-Mean-Square Deviation (RMSD), as well as the resulting root mean square fluctuations (RMSF) and principal component analysis (PCA) for all trajectory structures. The Visual Molecular Dynamics (VMD) program was used for visualization, the structures. VMD is designed to display and analyze proteins and nucleic acids [31]. For each of the 36 systems, 100 ns MD simulation was carried out, which generated 50,000 frames of trajectory data.

## 3. Results

### 3.1. Kinases and Phosphorylation Studies

These phosphorylation studies revealed several human protein kinases are likely to phosphorylate the nsps. Among these, glycogen synthase kinase 3 (GSK-3) is the enzyme predicted as phosphorylated among all the nsps (Table 2). GSK3 is a serine/threonine-protein kinase that phosphorylates the serine and threonine amino acids on its target substrate. GSK-3 has a major role in signaling pathways, cellular proliferation, migration, regulation, apoptosis, etc. [32]. We selected two phosphorylation sites from each nsps with the highest score. Nsp1 has two identified phosphorylation sites: SER 56 (AGGHSYGAD) and THR 72 (DELGTDPYE). Nsp2 contains two identified phosphorylation sites: THR 73 (YELQTPFEI) and SER 351 (KSILSPLYA). Nsp3 has two identified phosphorylation sites: SER 64 (MQVESDDYI) and THR 145 (VCVDTVRTN). Nsp4 has two identified phosphorylation sites: SER 81(QRGGSYTND) and SER455 (YKYFSGAMD). Nsp5 has two identified phosphorylation sites: SER 284 (TILGSALLE) and THR 292 (EDEFTPFDV). Nsp6 has two identified phosphorylation sites, namely T238(YFRLTLGVY) and T248(YLVSTQEFR). Nsp7-8-12 has two identified phosphorylation sites: SER 583 (VIGTSKFYG) and SER904 (NDNTSRYWE). Nsp9 (PDB:6W4B) has two identified phosphorylation sites namely SER9 (NNELSPVAL) and THR83 (FVTDTPKGP). Nsp10 has two identified phosphorylation sites: THR96 (QIPTTCAND) and THR109 (TLKNTVCTV). Nsp13 has two identified phosphorylation sites: SER150 (TFKLSYGIA) and SER 541(SSQGSEYDY). Nsp15 has two identified phosphorylation sites: SER 127 (IGVCSMTDI) and SER285 (RFKESPFEL). Nsp16-10 has two identified phosphorylation sites: SER132 (DLIISDMYD) and SER264 (TAVMSLKEG). All phosphorylation sites and sequences are represented in Table 2.

### 3.2. The Root-Mean-Square Deviation (RMSD) Plot

**Figure 3:**
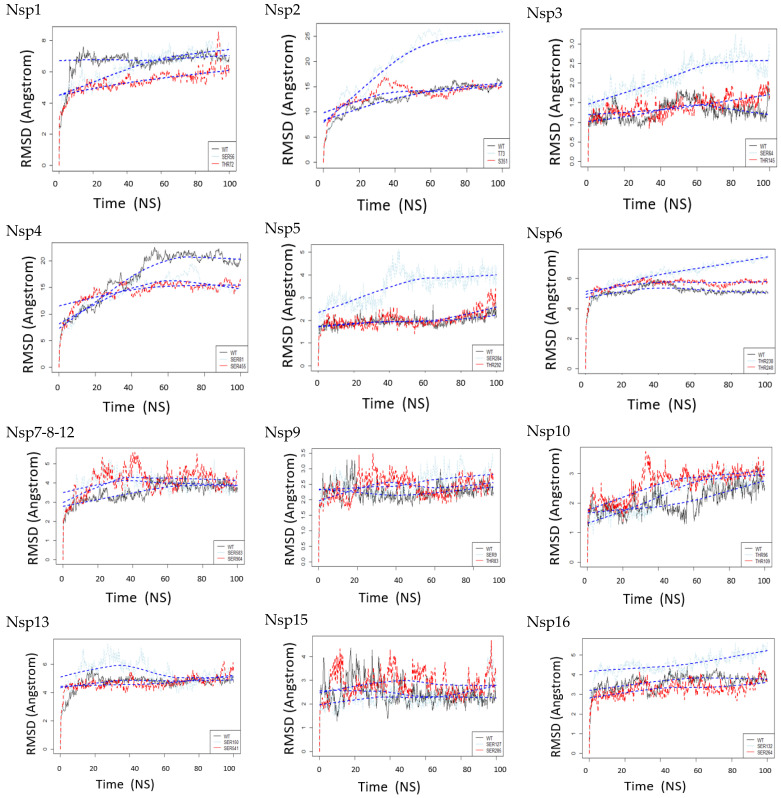
The RMSD of all residues for the 36 MD simulated structures (12 un-phosphorylated (Black), 12 phosphorylated sites 1 (Light blue), and 12 phosphorylated sites 2 (Red)) throughout the simulation (100 ns) compared to the starting confirmation (un-phosphorylated WT at time 0 ns) to measure the behavior effect of phosphorylation of 12 phosphorylated sites 1 and 12 phosphorylated sites 2 on the nsps.

To gain insight into the role of phosphorylation of both sites, we examined the structural effects of phosphorylated amino acid residues in the nsps. As the next step, we analyze trajectories of 100 ns MD simulation. The RMSD of all residues for the 36 MD simulated structures (12 un-phosphorylated, 12 phosphorylated sites 1, and 12 phosphorylated sites 2) throughout the simulation (100 ns) to explore the dynamic stability of the protein backbone during the simulation and measure the effect of phosphorylation of 12 phosphorylated sites 1 and 12 phosphorylated site 2 on the nsps behavior. Protein structures are more or less similar if the RMSD value is less than 1.2 angstroms since protein fluctuations might lead to significant deviation [33]. The RMSD from the initial structures for all 36 systems was calculated and plotted (Figure 3). The RMSD between the structures are shown in Table 3. For example, we find that nsp1 THR 72 (DELGTDPYE) phosphorylation shows significant conformational changes compared to the WT and nsp1 SER 56 which is predicted to be phosphorylated by CKII\GSK. When we compare the behavior with simulations of nsp2, the WT, and two phosphorylation sites, the nsp2 THR 73 (YELQTPFEI), which is predicted to be phosphorylated by p83MAPK\CKII\GSK3, have significant conformational changes compared to WT and another residue, SER 351, which did not show much fluctuation during MD studies. During the phosphorylation of nsp3 at SER 64 and THR 145, the RMSD value shows significant changes in SER 64, which is predicted to be phosphorylated by CKII\CaM-II\GSK3 more than WT and SER 145. Nsp4 SER 81 is predicted to be phosphorylated by PKA\AGC\DMPK\GEK, and SER455 have more significant changes than WT. Nsp5 SER 284, which is predicted to be phosphorylated by CKII\cdc2\CaM-II\GSK3, has significant changes compared to WT and THR 292. In nsp6 T238, which is predicted to be phosphorylated by PKC\PKA\GSK3\PKG\cdc2, the RMSD has significant changes compared to WT and T248. Nsp7-8-12 SER 583, which is predicted to be phosphorylated by CaM-II\GSK3\cdc2 and SER904, predicted to be phosphorylated by CKI\CKII\CaM-II the RMSD have significant changes, especially in the first 50 ns than WT. Nsp9 SER9 has been reported to have slightly more conformational change than its WT residue and THR83. Nsp10 has two phosphorylation sites (THR96 and THR109); neither showed any significant changes. Nsp13 SER150 RMSD has significant changes predicted to be phosphorylated by AGC\PKC\PKCa PRKCG\GSK3\cdc2 compared to WT. Nsp15 has two phosphorylation sites, SER 127 and SER285, and neither site showed significant changes. This means it may require longer simulation time. Nsp16 SER132 is predicted to be phosphorylated by CKII\GSK3\CaM-II\cdc2 compared to WT. Our results suggest that kinase inhibitors should be employed to explore these residues further in the future to determine their role in drug binding. Comparing MD simulations on WT and phosphorylated nsps enabled us to anticipate possible perturbations in the target structure and dynamics induced by phosphorylation at certain positions.

### 3.3. The Residue-Based Root Mean Square Fluctuation (RMSF)

**Figure 4:**
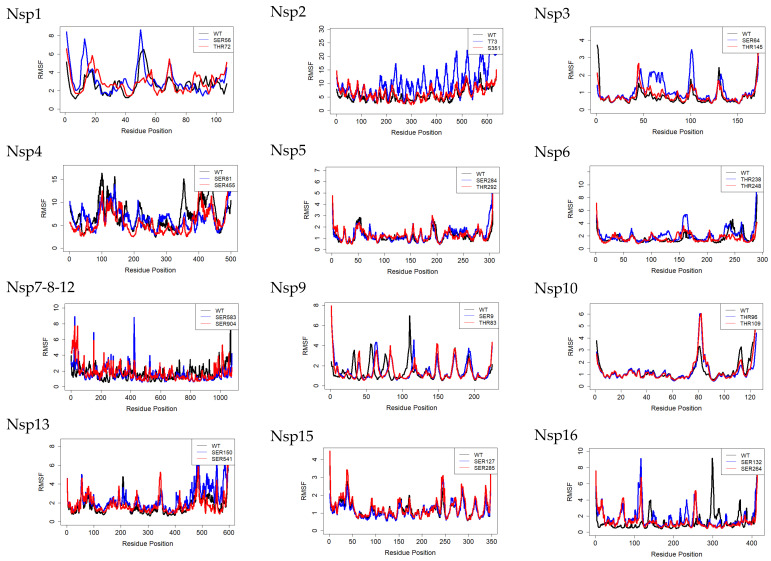
The RMSF measures the mobility of the protein backbone amino acid residues. We calculated RMSF for the 36 MD-simulated structures (12 un-phosphorylated, 12 phosphorylated sites 1, and 12 phosphorylated sites 2) throughout the simulation (100 ns throughout the simulation compared to the starting conformation.

The purpose of this study is to investigate whether phosphorylation enhances or affects the flexibility in each amino acid to determine a specific potential therapeutic target. The RMSF value can give us some insight by measuring the mobility of the protein backbone for each amino acid residue. Furthermore, we calculated RMSF for the 36 MD simulated structures (12 un-phosphorylated, 12 phosphorylated sites 1, and 12 phosphorylated sites 2) throughout the simulation, the plot shown in Figure 4. Higher RMSF value across the system indicates greater flexibility during the 100 ns molecular dynamics simulations. For the nsp1 SER 56 (AGGHSYGAD) and THR 72 (DELGTDPYE) RMSF plot, we found nsp1 SER 56 had more substantial fluctuations and an apparent increase in RMSF value of residues (more prominent in resides 80 to 90) than WT and THR 72. For nsp2, THR73 shows a higher value of RMSF than the wild type. The nsp15 wild type and phosphorylated regions show the same RSMF values.

## 4. Discussion

Changes in protein structure, including phosphorylation-related changes, significantly impact its function in general [34]. During SARS-CoV-2 infection the phosphorylation state of both host and viral proteins change dramatically [23,35]. Previous study have found 70 phosphorylation sites in SARS-CoV-2 proteins, including 5 in nsp3 and 1 each in nsp6, nsp9, nsp12, and nsp14 [35]. In our work, thirty-six SARS-CoV-2 systems were MD-simulated to untangle the possible effect of twenty predicted phosphorylation sites on the different nonstructural protein structures. The analyses showed significant structural deviations in the residues nsp1 THR 72, nsp2 THR 73, nsp3 SER 64, nsp4 SER 81, nsp4 SER 455, nsp5 SER284, nsp6 THR 238, and nsp16 SER 132.

SARS-CoV-2 nsps play a vital role in the virus’s life cycle and pathogenicity. Nsps have been identified as therapeutic targets in drug development research. A review by Raj et al. highlights the significance of each nsps, its function, and participation in drug research studies and other research [36]. Pharmacological inhibition of kinases has been shown to attenuate SARS-CoV-2 replication in host cells [23,37]. Despite the importance of the post-translational modifications, the underlying phosphorylation and replication mechanisms remain unclear in the case of SARS-CoV-2 [38]. In this regard, our in-silico study attempts to evaluate how phosphorylation influences protein’s conformational changes. Our investigation of protein phosphorylation studies using SARS-CoV-2 nsps revealed many potential candidate residue positions and corresponding human kinases in all selected algorithms (GPS 3.0, NetPhos3.1, and Scansite), along with their score and residue position in the sequences. The expectation is that phosphorylation of a functional phosphorylation site might cause a change in protein structure compared to the wild-type structure [39].. We used MD simulation to examine the effect of phosphorylation of residues. During simulation, we aim to explore global minima in the energy of proteins using a simulation time of 100 ns. Because of the limited computational resources running a triplicate was not an option. MD studies are a widely established technique for studying molecular evolution in biological systems, according to studies on COVID-19 [40,41,42].

Coronaviruses (α, β) encode nsp1, one of the first proteins to be expressed after cell entry to inhibit the host protein expression [43,44]. We find that nsp1 THR 72 (DELGTDPYE) phosphorylation shows significant conformational changes compared to the WT and nsp1 SER 56 which is predicted to be phosphorylated by CKII\GSK. Other studies have found that nsp1 suppresses innate immunity mainly by targeting type I interferon [45]. Recently, it has been shown that SARS-CoV-2 nsp1 causes translation inhibition by obstructing the entry region of the mRNA channel in the free 40S subunits and the non-translating 80S ribosomes [46]. The observed conformational change in the nsp1 THR 72 system may cause a similar sterical obstruction for the mRNA channel. The N-terminus (residues 1-116) have been observed to stabilize nsp1 binding to the ribosomal 40S subunit [47].

**Figure 5:**
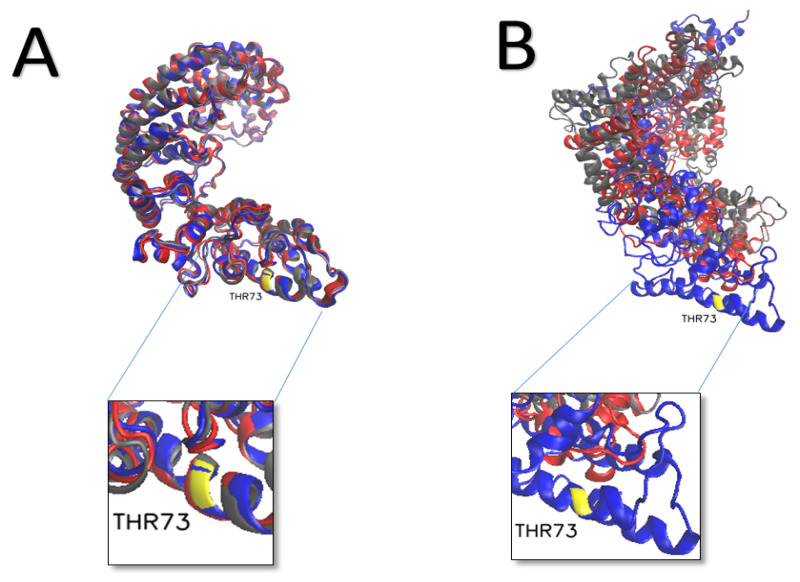
Changes in nsp2 structure with phosphorylation. The three simulated structures at time 100 ns (gray—WT, blue—T73, Red—S351). (**A**) at 0 nanoseconds; (**B**) after 100 nanoseconds.

Although fragments of nsp2 have been resolved experimentally, a full experimentally determined structure of nsp2 has not been determined [48]. In the N-terminal fragment (residues 1-288), three zinc finger binding domains that likely play a role in viral replication were observed [49]. Furthermore, nsp2 is thought to interact with many host proteins, such as EIF4E2 and GIGYF2, that form part of a complex repressing mRNA translation [50,51]. When we compare the behavior with simulations of nsp2, the WT, and two phosphorylation sites, the nsp2 THR 73 (YELQTPFEI), which is predicted to be phosphorylated by p83MAPK\CKII\GSK3, have significant conformational changes compared to WT and another residue, SER 351, which did not show much fluctuation during MD studies. Nsp2 is believed to disrupt the intracellular host signaling by interacting with host proteins prohibitin 1 (PHB1) and PHB2, which are implicated in several cellular functions, including cellular migration, differentiation, and apoptosis [52,53]. Deletion of the nsp2 coding sequence attenuates viral growth and RNA synthesis [54]. We find that nsp2 THR 73 (DELGTDPYE) phosphorylation shows significant conformational changes compared to the WT and nsp2 SER 351 (Figure 5).

**Figure 6:**
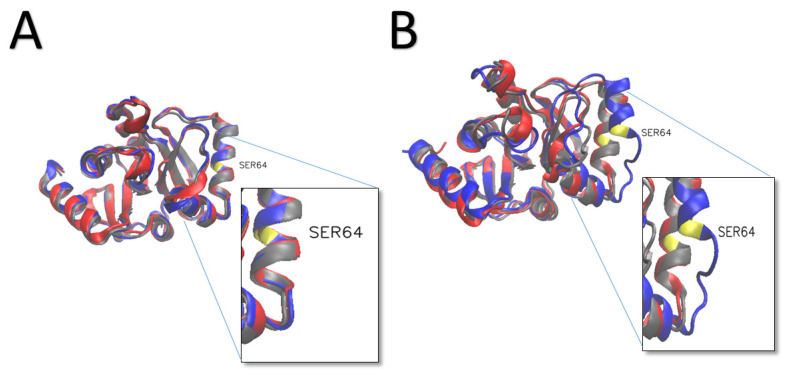
Changes in NSP3 structure with phosphorylation. The three simulated structures at time 100 ns (gray—WT, blue—S64, Red—T154). (**A**) at 0 nanoseconds; (**B**) after 100 nanoseconds.

Nsp3 is the biggest protein encoded by the coronavirus genome and plays many roles in the viral life cycle [55]. Based on phosphorylation prediction results, there were two significant hits for nsp3 residues: SER 64 predicted to be phosphorylated by CKII CaM-II GSK3 and THR 145 phosphorylated by kinase GSK3, PKC (Figure 6). Nsp3, nsp4, and nsp6 co-expression was shown to form double-membrane vesicles (DMVs) and convoluted membranes (CMs) [56]. Also, the interaction of nsp4 and the C-terminal of nsp3 (nsp3C) was observed in mouse hepatitis virus (MHV) [57]. Furthermore, the nsp3-nsp4 interaction has been recently found to be crucial for SARS-CoV replication through rearrangements of host-derived membranes [58]. Nsp3 SER 64, which falls in the ubiquitin-like domain 1 (Ubl1) domain of the protein which is related to single-stranded RNA (ssRNA) binding [59,60]. Thiscauses significant conformational changes compared to WT and nsp3 THR145 in our analysis. The amino-terminal ubiquitin-like domain of nsp3, which is considered to be essential for the virus, has been observed to interact with the N-protein C-terminal domain [61]. Furthermore, this interaction has been suggested as a potential for drug target [62]. 

Both nsp4 SER 81 and SER 455 show similar deviation from the WT trajectory, which a similar effect of both could explain. The amino acid residues 112–164 and 220–234 of nsp4 are necessary for the nsp3/nsp4 complex to cause host membrane rearrangement [58]. Furthermore, the amino acids residues 112–164 of nsp4 form the interaction domain with nsp3.

## 5. Conclusions

MD simulation studies are commonly used to determine the nature of molecules in biological systems. We investigated the structural changes in 16 SARS-CoV-2 virus nonstructural proteins caused by phosphorylation of computationally predicted sites in each protein on its behavior in a physiological environment. We found that the phosphorylation of nsp1 THR 72, nsp2 THR 73, nsp3 SER 64, nsp4 SER 81, nsp4 SER 455, nsp5 SER284, nsp6 THR 238, and nsp16 SER 132 show significant structural deviations from the WT trajectory as measured by RMSD. These identified locations may serve as candidates for further investigation in the future and may help identify potential drug targets.

## Figures and Tables

**Table 1:** Detailed descriptions of number of atoms and water box dimensions for each nonstructural protein (nsps) selected for the study.

Nonstructural Protein (Nsp)	Number of Atoms	Number of Atoms after Adding Water	Water Box Dimensions
Nsp1 Modeled	1630 atoms	24,413 atoms	56.2 Å × 63.2 Å × 61.7 Å
Nsp2 Modeled	9925 atoms	128,339 atoms	129.3 Å × 87.8 Å × 116.6 Å
Nsp3 PDB: 6WEY	3306 atoms	34,998 atoms	76.3 Å × 66.1 Å × 72.3 Å.
Nsp 4 Modeled	7889 atoms	168,068 atoms	140.5 Å × 93.9 Å × 131.6 Å
Nsp5 PDB: 6Y2E	4682 atoms	56,808 atoms	67.9 Å × 94.9 Å × 91.9 Å
Nsp6 Modeled	4606 atoms	48,099 atoms	67.8 Å × 79.7 Å × 92.8 Å
Nsp12-Nsp8-Nsp7 PDB: 7BW4	17,090 atoms	161,418 atoms	101.3 Å × 114.6 Å × 142.2 Å
Nsp9 PDB: 6W4B	3477 atoms	60,939 atoms	91.7 Å × 79.9 Å × 86.4 Å
Nsp10 PDB: 6ZCT	1801 atoms	29,080 atoms	62.9 Å × 67.5 Å × 71.4 Å
Nsp13 PDB: 6JYT	9467 atoms	118,438 atoms	110.8 Å × 105.5 Å × 104.3 Å
Nsp15 PDB 6VWW	2792 atoms	77,822 atoms	97.4 Å × 91.2 Å × 91.0 Å
Nsp16-14-10 PDB: 6W4H	6365 atoms	90,312 atoms	100.7 Å × 95.0 Å × 97.4 Å

**Table 2:** List of selected protein and phosphorylation sites and kinases.

Non-Structural Protein (Nsp)	Phosphorylated Residues	Site of Phosphorylation Shows the Sequence)	Potential Kinases
Nsp1 (107AA *, Modeled)	S56	AGGHSYGAD	GSK3
	T72	DELGTDPYE	CKII\GSK3
Nsp2 (638AA, Modeled)	T73	YELQTPFEI	p83MAPK\CKII\GSK3
	S351	KSILSPLYA	p83MAPK\JNK\cdc2\GSK3
Nsp3 (172AA, PDB: 6WEY)	S64-S269	MQVESDDYI	CKII\CaM-II\GSK3
	T145-S350	VCVDTVRTN	PKC\GSK3
Nsp 4 (400AA, Modeled)	S81	QRGGSYTND	PKA\AGC\DMPK\GEK
	S455	YKYFSGAMD	Unsp PKA PKC\GSK3
Nsp5 (306AA, PDB: 6Y2E)	S284--	TILGSALLE	CKII\cdc2\CaM-II\GSK3
	T292--	EDEFTPFDV	CKI\GSK3
Nsp6 (290AA, Model)	T238	YFRLTLGVY	PKC\PKA\GSK3\PKG\cdc2
	T248	YLVSTQEFR	DNAPK\GSK3\cdc2
Nsp12-8-7 (923AA, PDB: 7BW4)	S583-S592	VIGTSKFYG	CaM-II\GSK3\cdc2
	S904-S913	NDNTSRYWE	CKI\CKII\CaM-II
Nsp9 (117AA, PDB: 6W4B)	S9-S6	NNELSPVAL	Unsp\p38MAPK\cdk5
	T83-T80	FVTDTPKGP	Unsp\cdk5\PKC\GSK3\p38MAPK
Nsp10 (125AA, PDB: 6ZCT)	T96-T102	QIPTTCAND	GSK3\CaM-II\p38MAPK\CKII\CKI\cdc2
	T109-T115	TLKNTVCTV	CaM-II\PKA\GSK3\cdc2
Nsp13 (603AA, PDB: 6JYT)	S150-S184	TFKLSYGIA	AGC\PKC\PKCa\PRKCG\GSK3\cdc2\TBK
	S541-S539	SSQGSEYDY	CKI\CKII\GSK3\CaM-II\cdc2
Nsp15 (370AA, PDB: 6VWW)	S127-S104	IGVCSMTDI	PLK\PLK2\PLK2\CaM-II\CKII\GSK3
	S285-S262	RFKESPFEL	CMGC\DYRK\HIPK\HIPK2\CKII\GSK3\p83MAPK
Nsp16 (301AA, PDB: 6W4H)	S132-S6927	DLIISDMYD	CKII\GSK3\CaM-II\cdc2
	S264	TAVMSLKEG	AGC\PKC\PKCa\PRKCG\cdc2

* AA—amino acid residues.

**Table 3:** RMSD differences between wild type and phosphorylated structures.

		RMSD Å	
Protein	(WT-Mut1)	(WT-Mut2)	(Mut1-Mut2)
Nsp1	(WT-T72) 1.267	(WT-S56) 1.408	(S56-T72) 1.331
Nsp2	(WT-T73) 1.369	(WT-S351) 1.267	(T-73-S351) 1.334
Nsp3	(WT-T145) 0.940	(WT-S64) 0.949	(S64-T145) 1.052
Nsp4	(WT-S81) 1.471	(WT-S455) 1.502	(S81-S455) 0.977
Nsp5	(WT-S284) 0.941	(WT-S292) 1.265	(S284-S292) 0.905
Nsp6	(WT-T238) 1.252	(WT-T248) 1.186	(T238-T248) 1.104
Nsp7-8-12	(WT-S913) 1.278	(WT-S592) 1.300	(S913-S592) 1.229
Nsp9	(WT-S9) 0.836	(WT-T83) 0.919	(S9-T83) 1.125
Nsp10	(WT-T96) 1.095	(WT-T115) 0.962	(T96-T115) 1.042
Nsp13	(WT-S150) 1.086	(WT-S541) 1.255	(S150-S541) 1.128
Nsp15	(WT-S127) 1.095	(WT-S285) 1.288	(S127-S285) 1.185
Nsp16	(WE-S132) 1.146	(WE-S264) 0.952	(S132-S264) 1.25

## Data Availability

This paper doesn’t have any data to deposit to other resources as of now. All the result data associated with this paper is added as table and figures.
